# Cytokeratin 20‐Negative Merkel Cell Carcinoma: A Case Series and Discussion of Diagnostic Strategies

**DOI:** 10.1155/crdm/8439328

**Published:** 2025-12-29

**Authors:** Ryan H. Wealther, Kritin K. Verma, Ethan Matthew, Helen Chen, Cloyce Stetson

**Affiliations:** ^1^ Cook County Health Division of Dermatology, Chicago, Illinois, USA; ^2^ School of Medicine, Texas Tech University Health Sciences Center, Lubbock, Texas, USA, ttuhsc.edu; ^3^ Texas Tech University Health Sciences Center Department of Dermatology, Lubbock, Texas, USA

**Keywords:** CK20-negative, immunohistochemistry, Merkel cell carcinoma, pancytokeratin, small round blue cell tumors

## Abstract

Merkel cell carcinoma (MCC) is a rare, aggressive neuroendocrine carcinoma of the skin. MCC can present a diagnostic challenge, especially in cases where Cytokeratin 20 (CK20) is negative or focally positive. CK20‐negative MCC accounts for about 5% of MCCs. This case series describes five cases of CK20‐negative or focally positive MCC at an academic medical center. All cases were biopsies of cutaneous lesions. Histopathological investigation with hematoxylin and eosin staining revealed characteristic aggregates of small blue cell tumor morphology in all cases. CK20 staining was absent in three cases and focally positive in two, defined as less than 5% of tumor cells. All cases demonstrated pancytokeratin (PanCK) positivity in the paranuclear dot‐like pattern. Thyroid transcription factor 1 (TTF‐1) was negative in all instances, supporting the diagnosis of MCC. This case series illustrates the diagnostic value and limitations of PanCK and TTF‐1 staining in MCC in cases where CK20 is negative and discusses strategies for diagnosing this rare variant of MCC.

## 1. Introduction

Merkel cell carcinoma (MCC) is a rare and aggressive neuroendocrine skin cancer with increasing incidence, most often affecting elderly Caucasian males. MCC commonly arises on sun‐exposed sites, particularly in the head and neck region [1–5]. MCC grows rapidly and is often metastatic at the time of diagnosis, making a prompt and accurate diagnosis vital to initiating timely management for this deadly malignancy [1, 3, 5, 6].

On histological examination with hematoxylin and eosin (H&E) staining, MCC morphologically resembles other small round blue cell tumors such as metastatic small‐cell lung carcinoma (SCLC), basal cell carcinoma, melanoma, Ewing sarcoma, and lymphoma. Accurate diagnosis is critical yet challenging due to their morphological similarities [7, 8]. Importantly, MCC cannot be morphologically distinguished from metastatic small‐cell carcinoma (from the lung or elsewhere) by routine staining alone, and immunohistochemistry is essential for establishing the diagnosis [1, 7, 8]. Cytokeratin 20 (CK20) is frequently used to diagnose MCC as it is typically negative in other small round blue cell pathologies, including metastatic SCLC (negative in 94% of SCLC cases) [7–10]. CK20 is positive in roughly 87%–95% of MCC cases, classically in a paranuclear dot‐like pattern [7, 10].

CK20‐negative MCCs present a considerable diagnostic problem [7–10]. These uncommon variants, which account for around 5%–13% of all MCCs, necessitate alternative diagnostic methods [7, 8, 10, 11]. The immunohistochemical (IHC) workup for CK20‐negative MCC is complex and evolving, and there is no agreed‐upon algorithm for diagnosing these cases. IHC staining for pancytokeratin (PanCK) is often employed in MCC cases where CK20 is negative, as some sources have found PanCK in the paranuclear dot‐like staining pattern to be more specific for MCC than metastatic SCLC [12, 13]. However, other studies have shown that PanCK positivity in a paranuclear dot‐like pattern may be present in SCLC as well [14]. Nonetheless, PanCK in a paranuclear dot‐like pattern is one of the IHC stains recommended by the 2026 National Comprehensive Cancer Network (NCCN) Clinical Practice Guidelines In Oncology (NCCN Guidelines) for the diagnosis of MCC​ when CK20 is negative [15]. Additionally, thyroid transcription factor 1 (TTF‐1) is a useful IHC marker in the diagnosis of MCC, as it is generally positive in SCLC (88% positive) but negative in MCC (93% negative) [7, 10, 16]. Lastly, neurofilament is another IHC stain used in the diagnosis of MCC as it is positive in around 80% of MCCs and negative in most SCLCs (1%) [10]. Ultimately, the diagnosis of both CK20‐positive and CK20‐negative MCC relies on clinicopathologic correlation in the context of proper immunohistochemistry. A primary cutaneous small round blue cell tumor without evidence of metastatic disease from another organ, a positive CK20, and a negative TTF‐1 is sufficient for most dermatopathologists to diagnose MCC. When CK20 is negative, we consider PanCK in a paranuclear dot‐like pattern, a negative TTF‐1, and no evidence of metastatic disease to be sufficient to diagnose CK20‐negative MCC. This case series presents five CK20‐negative or focally positive CK20 MCCs and their associated histologic and IHC characteristics.

## 2. Cases

To identify cases, we searched dermatopathology staining records from an academic medical center from January 2000 to April 2024. Cases including stains for CK20 and PanCK (AE1/AE3) were selected for inclusion in the study. A total of 21 potential cases were identified. A dermatopathologist reviewed the cases’ histology and IHC staining and identified 5 cases that were consistent with CK20‐negative (or focally positive CK20) MCC. The original pathology reports were available with each of the five cases, which provided clinicopathologic correlation. Photographs of the slides were taken with the cellSense imaging software and an Olympus DP25 Microscope camera.

**Figure 1:**
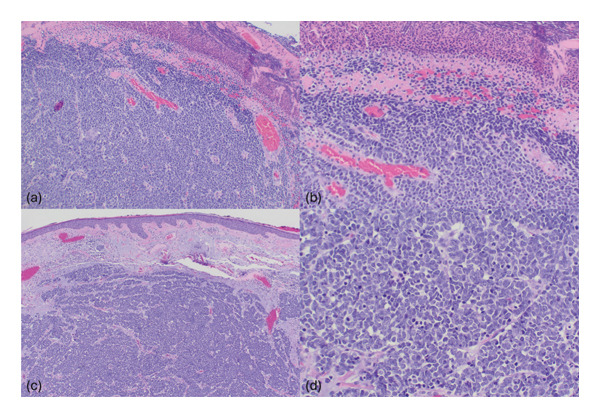
H&E staining of Cases 1 (a, 100×; b, 200×) and 2 (c, 40×; d 200×). Sections show micronodular aggregates of small blue cells. H&E: Hematoxylin and Eosin.

**Figure 2:**
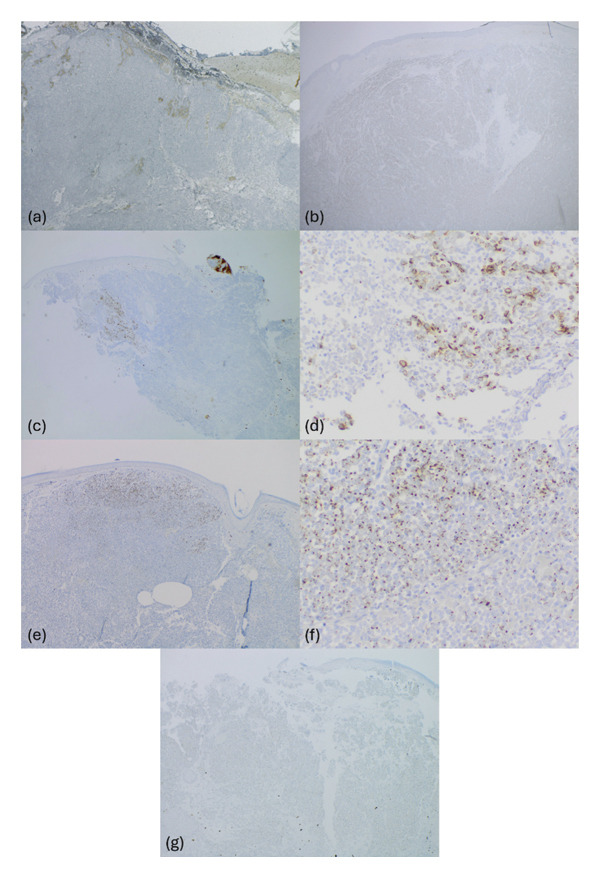
CK20 immunohistochemical staining of Case 1 (a, 20×), Case 2 (b, 20×), Case 3 (c, 100×; d, 200×), Case 4 (e, 40×; f, 200×), and Case 5 (g, 20×). Cases 1, 2, and 5 are negative for CK20, Case 3 is focally positive, and Case 4 demonstrates less than 5% positivity in the paranuclear dot‐like pattern. CK20: Cytokeratin 20.

**Figure 3:**
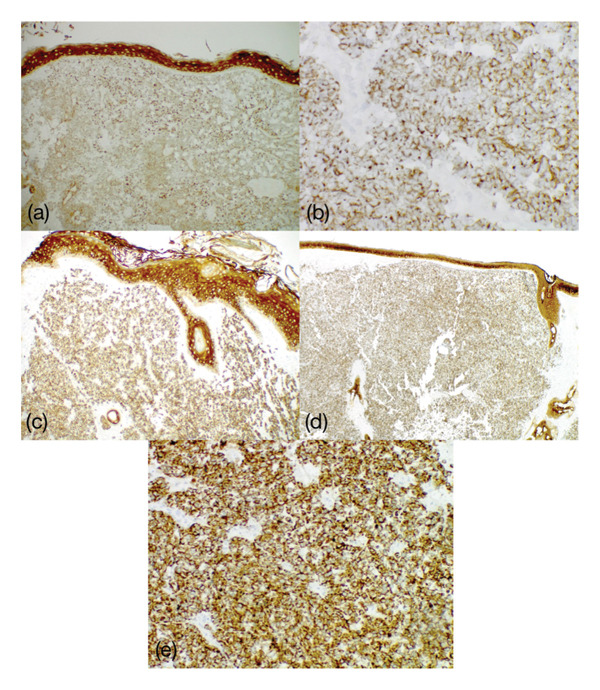
PanCK staining at 20× magnification of Cases 1 (a, 40×) and 2 (b, 400×), 3 (c, 100×), 4 (d, 40×), and 5 (e, 400×). Staining is strongly and diffusely positive in the paranuclear dot‐like pattern. PanCK: Pancytokeratin (AE1/AE3).

All five cases were biopsies of cutaneous lesions and consisted of aggregates of small blue cells on H&E staining, as seen in Figure 1 and summarized in Table 1. On IHC staining, three of the cases were completely negative for CK20 (Figure 2), while two of the cases exhibited focal staining of CK20 (stained less than 5% of the total tumor), prompting additional testing. All five cases were negative for TTF‐1 but were strongly and diffusely positive for PanCK in the paranuclear dot‐like pattern (Figure 3), which supported the diagnosis of MCC in all instances. Other stains were variably obtained and are summarized in Table 1.

**Table 1:** A summary of clinical, histologic, and immunohistochemical findings of each of the five cases of CK20‐negative MCCs.

Case number	Clinical history from biopsy report	H&E	CK20	PanCK	TTF‐1	SOX‐10	S‐100	LCA
1	13 mm heme‐crusted friable nodule on the right temple	Micronodular aggregates of small blue cells	Negative	Strongly and diffusely positive in the paranuclear dot‐like pattern	Negative	N/A	N/A	N/A
2	2.1 × 1.8 cm pink erythematous nodular plaque on the left upper chest	Micronodular aggregates of small blue cells	Negative	Strongly positive with the paranuclear dot‐like pattern	Negative	Negative	N/A	N/A
3	Friable nodule on the left external auditory canal	Dermis infiltrated by atypical small to intermediate blue cells with numerous mitoses	Less than 5% stained with paranuclear dot‐like pattern	Strongly and diffusely positive in the paranuclear dot‐like pattern	Negative	N/A	N/A	Negative
4	9 mm brown nodule on the left parietal scalp that started as a “freckle” 3 months ago and has grown	Small to intermediate blue cell tumor filling the dermis	Focal paranuclear dot‐like pattern, negative in the majority of tumor	Strongly and diffusely positive in the paranuclear dot‐like pattern	Negative	N/A	Negative	N/A
5	Shave biopsy, right angle of jaw	Full thickness atypia of keratinocytes in epidermis with malignant small blue cells infiltrating the dermis	Negative	Strongly and diffusely positive in the paranuclear dot‐like pattern	Negative	N/A	N/A	N/A

Abbreviations: CK20, Cytokeratin 20; H&E, Hematoxylin and Eosin; LCA, leukocyte common antigen (CD45); N/A, not applicable; PanCK, pancytokeratin (AE1/AE3 in PanCK); S‐100, S‐100 protein; SOX‐10, SRY‐Box transcription factor 10; TTF‐1, thyroid transcription factor 1.

## 3. Discussion

CK20‐negative MCCs present a diagnostic challenge. The combination of positive CK20 and negative TTF‐1 is generally considered the current standard for diagnosing MCC in cutaneous tumors consisting of aggregates of small blue cells [7, 15–17]. As discussed earlier, the diagnosis of CK20‐negative MCC is more complex. Commonly employed IHC stains used in the diagnosis of CK20‐negative MCC include neurofilament, chromogranin, synaptophysin, CD56, neuron‐specific enolase (NSE), Merkel cell polyomavirus large T antigen, special AT‐rich sequence‐binding Protein 2 (SATB2), paired Box 5 (PAX5), cytokeratin CAM 5.2, and AE1/AE3 [7, 8, 10–13]. In the five cases presented, appropriate clinical context, H&E consistent with MCC, negative or focally positive CK20 staining, negative TTF‐1, and the characteristic paranuclear dot‐like pattern of PanCK contributed to the diagnosis of MCC.

**Figure 4:**
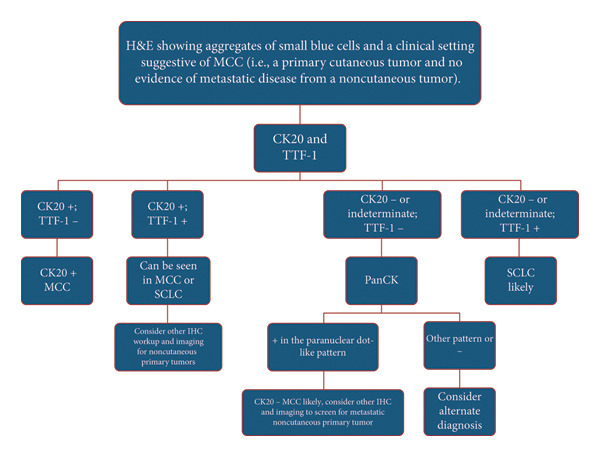
A diagnostic algorithm for diagnosing CK20‐negative or indeterminate MCC. CK20: Cytokeratin 20, H&E: Hematoxylin and Eosin, MCC: Merkel cell carcinoma, PanCK: pancytokeratin, SCLC: small‐cell lung carcinoma, TTF‐1: thyroid transcription factor 1.

In MCC, H&E reveals aggregates of small blue cells in the dermis, which are commonly organized in sheets or trabecular arrays [1]. IHC is critical in distinguishing MCC from other small blue cell pathologies [1, 7, 8, 10]. The following is the diagnostic strategy our institution uses for diagnosing MCC (Figure 4), which is consistent with the 2026 NCCN Guidelines for diagnosing MCC [15]. Following initial H&E staining suggestive of MCC with the appropriate clinical context (i.e., a primary cutaneous tumor without evidence of metastatic disease from a noncutaneous primary tumor), IHC staining for CK20 and TTF‐1 is obtained. If CK20 is positive and TTF‐1 is negative, the diagnosis of MCC can be made. In CK20‐negative or indeterminate cases, additional IHC staining for PanCK is obtained [12, 13, 15]. Positivity of PanCK in the paranuclear dot‐like pattern with a concurrent negative TTF‐1 in the appropriate clinical context suggests a diagnosis of CK20‐negative MCC [7, 10, 12, 13, 15]. Clinical and radiographic screening to exclude metastasis from primary noncutaneous small cell carcinoma (e.g., SCLC) and other IHC stains for MCC (e.g., neurofilament, chromogranin A, synaptophysin, and Merkel cell polyomavirus large T antigen) can be obtained if the diagnosis remains in question [7, 10, 15, 17]. This method can distinguish MCC from other neuroendocrine tumors, particularly SCLC [7, 12, 13, 15]. Lastly, the diagnostic algorithm of CK20‐negative MCC is not firmly established, and given the limitations of TTF‐1 and PanCK, clinicians will have varied diagnostic approaches that may include other diagnostic workup, including additional IHC, clinicopathologic correlation, and imaging.

Our study’s shortcomings include its retrospective nature and limited sample size, as only five cases of CK20‐negative (or CK20 focally positive) MCCs were identified within nearly 24 years’ worth of cases. Additionally, the lack of access to our cases’ demographic and clinical histories remains another limitation of our study. However, considering the rarity of CK20‐negative MCCs, even small case series can provide useful information about diagnostic techniques. Our case series demonstrates that PanCK staining with a paranuclear dot‐like pattern paired with negative TTF‐1 staining within the appropriate clinical context can be an effective technique for identifying CK20‐negative MCCs. CK20‐negative MCC remains difficult to diagnose, and more research is needed to identify a universal approach for diagnosing this rare and deadly disease.

## Patient Perspective

No written consent has been obtained from the patients as there are no patient identifiable data included in this case series.

## Conflicts of Interest

The authors declare no conflicts of interest.

## Funding

No funding was received for this manuscript.
